# Regulation of Slow and Fast Muscle Myofibrillogenesis by Wnt/β-Catenin and Myostatin Signaling

**DOI:** 10.1371/journal.pone.0005880

**Published:** 2009-06-11

**Authors:** Jin-Ming Tee, Carina van Rooijen, Rick Boonen, Danica Zivkovic

**Affiliations:** Hubrecht Institute for Developmental Biology and Stem Cell Research and University Medical Center, Utrecht, The Netherlands; McMaster University, Canada

## Abstract

Deviation from proper muscle development or homeostasis results in various myopathic conditions. Employing genetic as well as chemical intervention, we provide evidence that a tight regulation of Wnt/β-catenin signaling is essential for muscle fiber growth and maintenance. In zebrafish embryos, gain-of-Wnt/β-catenin function results in unscheduled muscle progenitor proliferation, leading to slow and fast muscle hypertrophy accompanied by fast muscle degeneration. The effects of Wnt/β-catenin signaling on fast muscle hypertrophy were rescued by misexpression of Myostatin or p21^CIP/WAF^, establishing an *in vivo* regulation of myofibrillogenesis by Wnt/β-catenin signaling and Myostatin. Epistatic analyses suggest a possible genetic interaction between Wnt/β-catenin and Myostatin in regulation of slow and fast twitch muscle myofibrillogenesis.

## Introduction

Understanding muscle development is crucial for generating novel regenerative therapies for muscle diseases and treating muscle injuries. Extensive research has contributed to the current understanding of various aspects of somitogenesis and myogenesis. The periodicity of rostral-caudal somite formation [1] as well as their differentiation into the axial skeleton, skeletal muscle and dorsal dermis are similar in all vertebrates [2]. Furthermore, the zebrafish determinate muscle growth by hyperplasia, the increase in muscle fiber number, and by hypertrophy, the increase in muscle fiber size, are comparable to mammalian muscle growth making it a suitable model system to study myofibrillogenesis and various myopathies [3]–[5].

Wnt/β-catenin pathway plays a crucial role in early somitogenesis and myogenesis in birds [6], [7], mice [8]–[10] as well as in zebrafish [11] by affecting skeletal muscle development at several levels, including mesodermal patterning, segmentation clock and myoblast differentiation [12], [13]. The Wnt/β-catenin signaling regulates Lef/Tcf–mediated transcription of downstream target genes via the transcriptional coactivator β-catenin [14]. In the absence of Wnt ligand, β-catenin is targeted for proteosomal degradation by a “destruction complex” comprising of CK1, GSK3β, Axin1 and Apc1. The fine balance between proliferation and differentiation required for proper development and growth of the myotome depends on signaling cues originating from tissues surrounding the somites [15], [16], including Wnt ligands. Cumulative evidence implicates Hedgehog and Fgf8 signaling in specification and differentiation of slow and fast twitch muscle fibers respectively, during the first wave of myogenesis [17]–[20]. Although recent work has shown the role of Hedgehog signaling in differentiation of a subset of secondary slow twitch muscle fibers [19], the precise molecular mechanism underlying specification and maintenance of secondary fast twitch muscle fibers as well as the Hedgehog independent slow twitch muscle fibers remains to be elucidated.

This study shows that upward deviation from the tightly controlled physiological level of Wnt/β-catenin activity by genetic and chemical intervention in zebrafish embryos leads to compromised growth and maintenance of slow and fast muscle fibers. This phenotype derives from hyperproliferation of the Pax3/7+ pre-myogenic precursors. Hence, misexpression of p21^CIP/WAF^ or *mstn* in the embryos with gain-of-Wnt/β-catenin function restores the integrity as well as morphology of the fast muscle fibers. We further discuss the possibility that this tight and opposing regulation of myofibrillogenesis by Wnt/β-catenin and Myostatin in zebrafish could operate through their genetic interaction.

## Results

### Wnt/β-catenin hyperactivity causes loss of somites and aberrant muscle fibers

**Figure 1:**
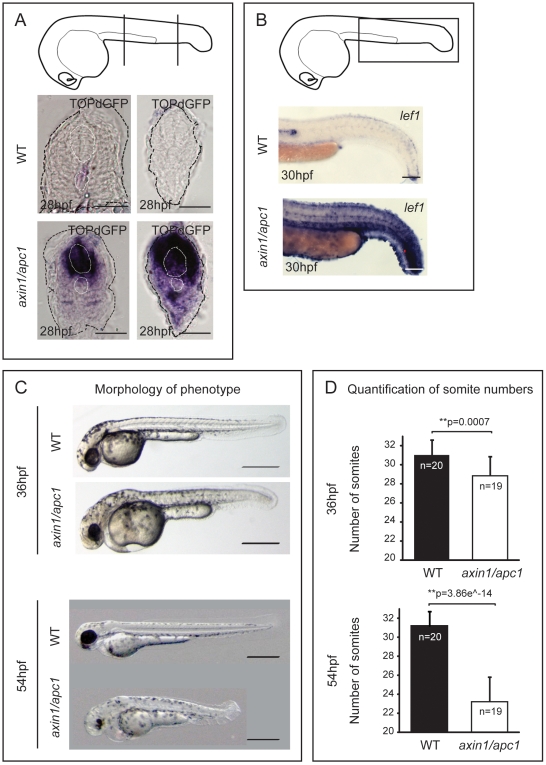
Hyperactivation of Wnt/β-catenin pathway leads to late somite-loss. (A) TOPdGFP transgenic embryos report activated Wnt/β-catenin signaling, i.e. TOPdGFP transcripts. Cartoon depicts the level of vibratome sectioning i.e. left panel at the yolk extension and right panel caudal to the yolk extension. Scale bar, 50 µm. (B) Hyperactivation of a target of Wnt/β-catenin pathway *lef1*, as shown with WISH, in *axin1/apc1* mutants matches the expression of TOPdGFP. Scale bar, 250 µm. (C) The *axin1/apc1* embryos are slightly shorter compared to wild-type embryos at 36 hpf, top panels. At 54 hpf, the difference becomes striking, bottom panels. Scale bar, 500 µm. (D) Somite counts at 36 hpf and 54 hpf, corresponding to embryos depicted in (C) with error bars showing the standard deviation.

Wnt/β-catenin gradient has been shown to be important for somite segmentation [9]. Importantly, it has been suggested that Wnt/β-catenin is downregulated in the somite following skeletal muscle differentiation [21]. We investigated the expression of Wnt/β-catenin reporter TOPdGFP [22] during post-segmentation corresponding to the second wave of myogenesis. Consistent with previous studies [21], we observed only faint expression in the trunk and tail of wild-types at 28 hours post-fertilization (hpf) (Fig. 1A). Next, by employing the homozygous compound zebrafish mutants of *axin1* [23], [24] and *apc1* [25] (hereafter referred to as *axin1/apc1*), we investigated whether Wnt/β-catenin is hyperactivated in the somites. Indeed, there was strong ectopic expression of the TOPdGFP reporter in *axin1/apc1* mutants in a rostro-caudally rising gradient (Fig. 1A). This corresponded to enhanced expression of the Wnt transcription factor and its direct target gene *lef1* [26] throughout the somites (Fig. 1B), showing that Wnt pathway is overactivated in the somites of *axin1/apc1* mutants.

At completion of segmentation and the first wave of myogenesis at 24 hpf, *axin1/apc1* embryos had a normal number of somites, size of somites (Fig. S1), as well as normal muscle fiber formation (data not shown). The earliest clear somite phenotype in *axin1/apc1* mutants was at 36 hpf, with a slight decrease in somite number from approximately 31 to 29 (Fig. 1, C and D). Strikingly, at 54 hpf, there was a severe tail truncation due to loss of approximately 10 somites (Fig. 1, C and D). The formation of normal somites at 24 hpf (Fig. S1) eliminates somite fusion and abnormal initiation of segmentation as an underlying cause of somite loss. Hence, this late and gradual somite-loss strongly suggests that the underlying mechanism does not entail a defect in somite induction and/or patterning.

**Figure 2:**
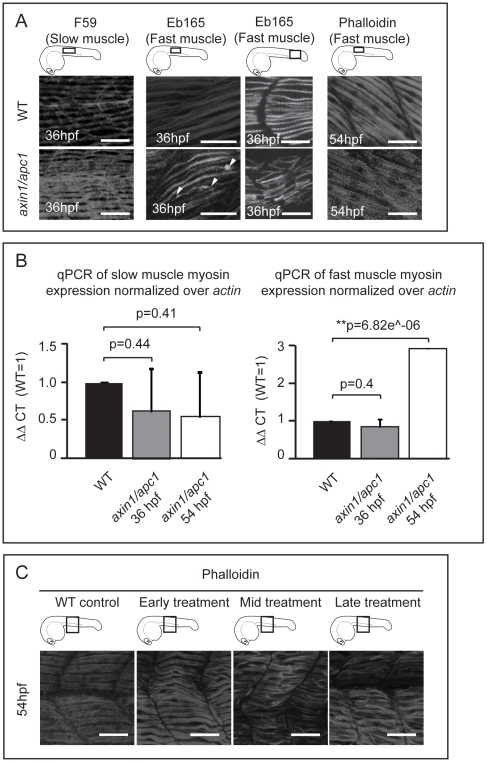
Late hyperactivation of Wnt/β-catenin pathway leads to aberrant myofibrillogenesis. (A) *axin/apc1* embryos at 36 hpf have thickened slow muscle fibers, left panels. The fast muscle fibers at 36 hpf are detached from the vertical myoseptum, forming lesions (white arrow heads) and are disorganized, middle panels. At 54 hpf, fast muscle fibers are thickened (hypertrophic), right panels. All embryos were imaged at the positions as depicted in cartoons. Images for F59 and Eb165 at 36 hpf are cumulative z-stacks. Images for Phalloidin at 54 hpf are single z-plane at the level of fast muscle fibers. Scale bar, 25 µm. (B) Quantitative real-time PCR (qRT-PCR) of *myhz2* (fast muscle specific) and *myhz5* (slow muscle specific) mRNA expression normalized to *actin*. Graphs show that the quantity of *myhz5* is not significantly different in *axin1/apc1* embryos, failing to identify subtle difference as shown in Fig. 2A. The quantity of *myhz2* is upregulated in *axin1/apc1* embryos at 54 hpf. (C) LiCl treatment during various time intervals. Early treatment = tailbud stage and 16 hpf for 40 minutes each on the same clutch of embryos, mid treatment = at 16 hpf and 24 hpf and late treatment = at 24 hpf and 30 hpf. Embryos were stained with Phalloidin for visualization of all muscle fibers. All embryos were imaged at the level of the yolk extension, as depicted in cartoon. Images are cumulative z-stacks. Scale bar, 25 µm.

Next, we examined whether upregulation of the Wnt/β-catenin signaling would affect the fast- and slow-twitch muscles that make up the myotome. The slow muscle fibers appeared to be hypertrophic, as well as hyperplastic with an additional 2–4 fibers per somite (n = 4) (Fig. 2A). Strikingly, the fast muscle fibers were disorganized, with some muscle fibers detaching from the vertical myoseptum, forming small lesions, while becoming hypertrophic only at 54 hpf (Fig. 2A). Confirming the distinct effects of hyperactive Wnt/β-catenin on slow versus fast muscle fibers, quantification by RT-qPCR of myosin heavy chain specific for slow or fast twitch muscle fibers revealed an increase in fast muscle myosin at 54 hpf (Fig. 2B). However, there is no significant difference observed for slow muscle myosin (Fig. 2B).

We confirmed the specific role of Wnt/β-catenin by employing a chemical activator of the Wnt/β-catenin pathway, lithium chloride (LiCl). The wild-type embryos treated with LiCl prior to completion of somitogenesis, at tailbud and mid-somitogenesis were truncated and curled albeit no detached muscle fibers were present (Fig. 2C, early treatment). In contrast, the embryos treated after 24 hpf showed severe muscle fiber detachment and hypertrophy (Fig. 2C, mid-treatment and late-treatment), resembling *axin1/apc1* mutants. Hence, the muscle fiber defect in the *axin1/apc1* mutants is likely also caused by late Wnt/β-catenin hyperactivation.

**Figure 3:**
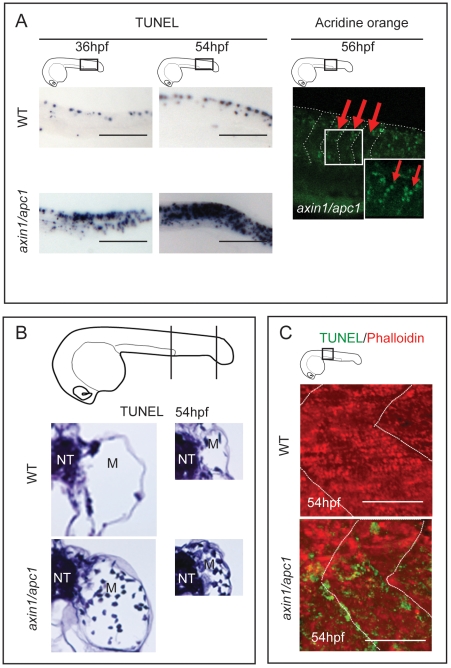
Apoptosis at somite boundaries and muscle fibers. (A) From 36 hpf onwards, *axin1/apc1* embryos show an increase in apoptosis as labeled by TUNEL. Insets show the imaged area. Scale bar, 100 µm. Apoptotic cells labeled with acridine orange lining up at the somites boundaries, right panel. Arrows and lines mark the somite boundaries. Scale bar, 25 µm. (B) TUNEL labeling at 54 hpf show an increase in apoptotic cells in the myotome. Cartoon depicts the level of sectioning i.e. left panel at the posterior end of the yolk extension and right panel posterior end of the tail. NT-neural tube; M-myotome. (C) Co-labeling of fluorescent TUNEL assay (apoptotic cells) and phalloidin labeling (muscle fibers), left and middle panel. This CLSM image was taken caudal to the yolk extension, at a single z-plane of 5 µm of fast muscle fibers.

The fast muscle fiber degeneration beginning at 36 hpf, corresponded with gradual increase in apoptosis (Fig. 3A), with apoptosis occurring within the myotome (Fig. 3B) and along the vertical myoseptum (Fig. 3, A and C).

### Hyperactive Wnt/β-catenin drives muscle progenitors into unscheduled proliferation

**Figure 4:**
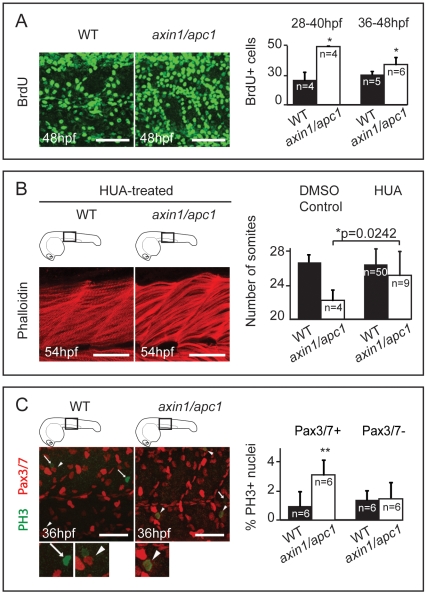
Myotome hyperproliferation and sustained differentiation in *axin/apc1* embryos. (A) BrdU pulse was performed at 36 hpf, chased for 12 hours, and imaged at 48 hpf. Embryos were imaged at the level of the yolk extension. Scale bar, 25 µm. BrdU+ pulse was performed at 28 hpf or 36 hpf, and quantification of number of BrdU+ proliferating cells per somite was done 12 hours later at 40 hpf or 48 hpf, respectively. (C) HUA treatment of embryos from 24 hpf until fixation at 54 hpf. Inhibition of proliferation with HUA from 24 hpf results in rescue of muscle hypertrophy. Muscle fibers were stained with Phalloidin, and imaged at 54 hpf at the level of the yolk extension. Compare with untreated wild-types in Fig. 2A (right panels). Scale bar, 25 µm. Quantification of number of somites is increased in *axin1/apc1* mutants upon HUA treatment. (E) Colocalization of Pax3/7+ and PH 3+ cells shows proliferating muscle progenitors. Quantification of proliferating Pax3/7+ and Pax3/7- cells in the wild-types vs. *axin1/apc1* mutant embryos shows significantly more proliferating Pax3/7+ cells in the mutants. Scale bar, 50 µm.

The ability of Wnts to enhance proliferation in the dermomyotome [27] led us to hypothesize that unscheduled proliferation in the somites might lead to muscle hypertrophy in *axin1/apc1* mutants. While at 16 hpf there was no difference in proliferation between mutants and wildtypes (data not shown), from 28 hpf onwards, BrdU pulse experiments identified a sharp increase in number of cells in S-phase (Fig. 4A), which was confirmed by increased labeling of phosphohistone H3 (PH 3)+ mitotic cells (data not shown) and their quantification by FACS (Fig. S2B). To investigate whether this unscheduled increased proliferation caused muscle hypertrophy, we partially inhibited cell division with a combination of aphidicolin [28] and hydroxyurea (HUA) [29] from 24 hpf until fixation at 54 hpf. Strikingly, the fast muscle hypertrophy (Fig. 4B; compare to Fig. 2A, right panels) and degeneration (Fig. S3A) as well as the number of somites were partially rescued (Fig. 4B) confirming that hyperproliferation leads to the fast muscle hypertrophy and degeneration.

Pre-myogenic embryonic muscle progenitor cells expressing Pax3/7 transcription factors give rise to myoD+ myoblasts, myogenin+ myocytes and myotubes, that terminally differentiate into muscle fibers [30]. To determine whether these Pax3/7+ progenitors were stimulated by Wnt/β-catenin to hyper-proliferate, we performed co-labeling of PH 3 and Pax3/7 in the *axin1/apc1* mutants. There was a significant increase in co-localization of PH 3+ mitotic nuclei and Pax3/7+ nuclei in the *axin1/apc1* mutants (Fig. 4C) as compared to wild-types, suggesting that Wnt/β-catenin drives unscheduled proliferation of pre-myogenic progenitors. Surprisingly, the absolute number of Pax3/7+ cells was unaltered in *axin1/apc1* mutants (data not shown), suggesting that the newly born progeny of dividing Pax3/7+ cells was not maintained in a Pax3/7+ precursor state, but instead was instructed to differentiate. Hence, we examined myogenic differentiation in the mutants. Consistent with the unperturbed Pax3/7+ muscle progenitors at 16 hpf, *myoD* was unaltered in *axin1/apc1* mutants during initial myogenesis (data not shown). Importantly, later in development, its timely downregulation failed and its expression was sustained (Fig. S3B). Consistent with prolonged *myoD* expression in mutants, *myogenin* expression was also extended in *axin1/apc1* (Fig. S3C). As myoD+ myoblasts are known to proliferate it is possible that the ectopic and extended myoblast maintenance also contributes to the hyperproliferation in the mutants. To test this, we performed co-labeling of anti-MyoD with anti-BrdU antibody (with BrdU incorporation chased for 2 hours). We observed substantial increase in MyoD+ cells in *axin1/apc1* embryos (Fig. S3D), confirming the increased *myoD* RNA expression in the mutants. However, we do not observe an increase in proliferating (BrdU+) myoD+ cells (data not shown). The data is consistent with a positive role of Wnt/β-catenin signaling in driving myogenic differentiation [13].

### Myotomal proliferation and hypertrophy in axin/apc mutants are counteracted by misexpression of Mstn and its downstream target p21^CIP/WAF^

**Figure 5:**
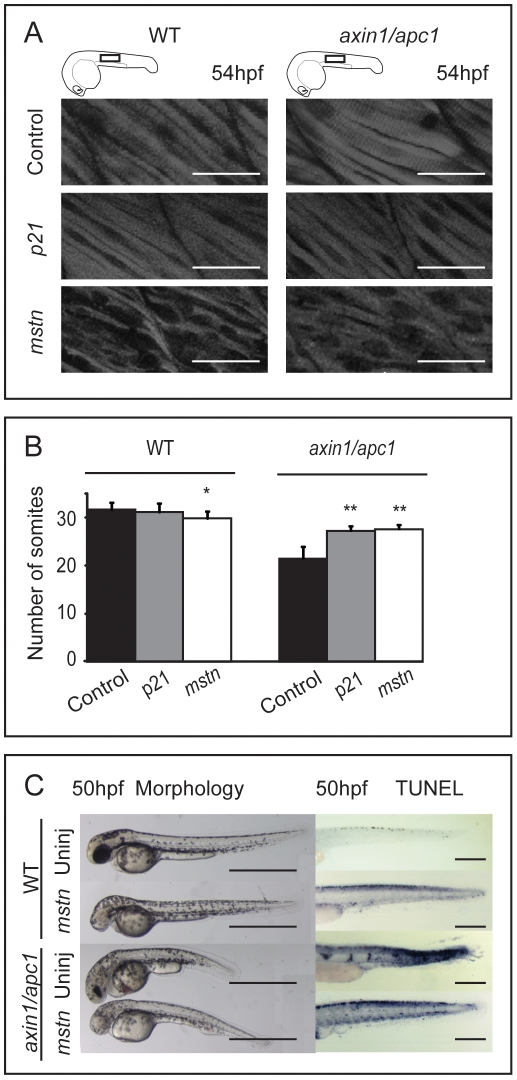
Misexpression of *mstn* rescues *axin1/apc1* embryos. (A) Injection of 5 pg p21^CIP/WAF^ or *mstn* mRNA into 1 cell stage embryos, and phenotype assessment at 54 hpf. Slightly hypotrophic muscle fibers are observed in *p21* as well as *mstn*-injected wild-type embryos confirming efficiency of misexpression. Muscle hypertrophy in *mstn*-injected *axin1/apc1* embryos is partially restored to normal. (B) Quantification of the somite number of uninjected and p21^CIP/WAF^ or *mstn* mRNA (5 pg) injected embryos. (C) Misexpression of *mstn* partially restores the truncated somite phenotype, as well as cell survival in *axin1/apc1*. Scale bar, 0.5 mm.

Reportedly, Wnt/β-catenin through its direct target *c-myc*, can downregulate p21^CIP/WAF^ (also known as cyclin-dependent kinase inhibitor 1A) [*31*]. We hypothesized that sustained myotomal proliferation in *axin1/apc1* mutants works through Wnt/β-catenin-mediated inhibition of p21^CIP/WAF^. We tested the hypothesis by examining the capacity of p21^CIP/WAF^ mRNA injected into *axin1/apc1* mutant to rescue muscle fiber phenotype. Employing misexpression with p21^CIP/WAF^ mRNA concentration that only subtly affected the wildtypes, muscle fiber hypertrophy was rescued in injected *axin1/apc1* embryos (Fig. 5A), suggesting that muscle fiber degeneration is due to hyperproliferation caused by failure of timely p21^CIP/WAF^-dependent cell cycle exit. However, we cannot exclude the possibility that forced cell cycle exit mediated by *p21^CIP/WAF^* misexpression in itself, and independently of its postulated positioning downstream of the Wnt pathway, may have brought about the rescue.

p21^CIP/WAF^ has been implicated in muscle differentiation as the downstream target of Mstn, a TGF-β family member. Mstn is a key negative regulator of muscle growth that promotes terminal differentiation of embryonic muscle progenitors through the activation of p21^CIP/WAF^ [32]. Decreased levels of Mstn, context dependently lead to muscle hypertrophy [32]. Based on the hypertrophic muscle phenotype in the compound mutants and upon late LiCl treatment of wildtype embryos, we hypothesize that p21^CIP/WAF^ may be epistatic to Mstn and that overactive Wnt pathway may through inhibition of Mstn downregulate p21^CIP/WAF^. We tested this possibility by performing rescue of muscle fiber degeneration in the *axin1/apc1* mutants via misexpression of *mstn* mRNA. While at this particular concentration of *mstn*, wild-types showed slightly hypotrophic muscle fibers, the *axin1/apc1* embryos showed a partial rescue of the hypertrophic muscle fibers (Fig. 5A). Consistently, misexpression of *mstn* rescued the number of somites (n = 8) and length of mutant embryos (Fig. 5, B and C). Importantly, cell survival in *axin1/apc1* mutants was also rescued (Fig. 5C, right panels).

**Figure 6:**
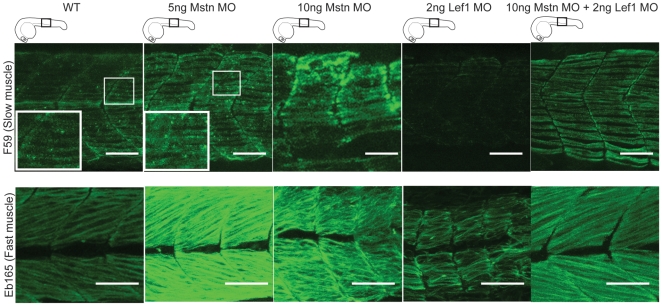
Mstn is dominant over Wnt/β-catenin in myofibrillogenesis. Representative images of injection of 5 ng (n = 30) or 10 ng (n = 30) Mstn MO results in muscle hypertrophy, whereas injection of 2 ng Lef1 MO (n = 30) results in hypotrophic muscle fibers. Co-injection of 10 ng Mstn MO with 2 ng Lef1 MO (n = 10) results in hypertrophic muscle fibers. Images of all embryos are cumulative z-stacks and taken at the level of the yolk extension, as depicted in cartoons. Scale bar, 50 µm.

To further explore opposing effects of Wnt/β-catenin and Myostatin on phenotypic aspects of myofibrillogenesis we asked whether morpholino (MO)-mediated knock-down of Mstn would result in a similar hypertrophic phenotype as hyperactive Wnt/β-catenin in zebrafish embryos. Injection of 5 ng *mstn* MO resulted in hypertrophic muscle fibers, while injection of 2 ng Lef1 MO resulted in hypotrophic muscle fibers (Fig. 6). We further co-injected 2 ng Lef1 MO with 10 ng Mstn MO, and asked whether muscle fibers would be hypertrophic or hypotrophic. The slow and fast muscle fibers appear hypertrophic (Fig. 6). As a loss of Mstn signaling would be expected to lead to hyperproliferation, and loss of Wnt/β-catenin signaling to reduction of proliferation of pre-myogenic precursors, the data suggests that loss of Mstn is dominant over the loss of Wnt/β-catenin signaling.

**Figure 7:**
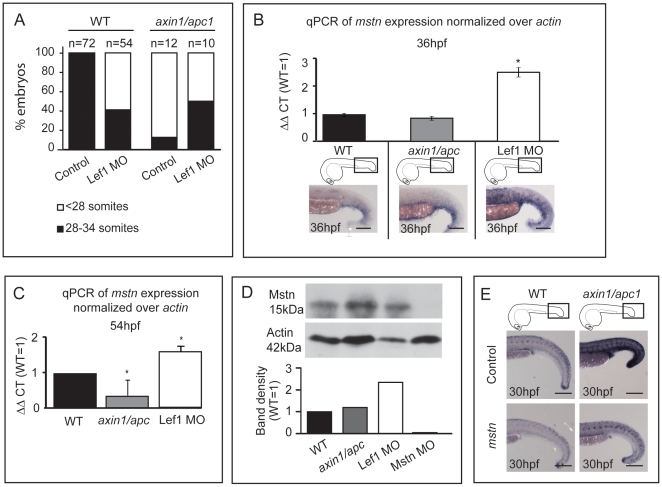
*mstn* is upregulated in LOF Wnt/β-catenin. (A) 2 ng Lef1 MO was injected at 1-cell stage into wild-type or *axin1/apc1* embryos, and the number of somites was counted at 54 hpf. Two independent clutches of *axin1/apc1* heterozygous incross were analyzed (Total n = 64), in which 10 were genotyped as *axin1/apc1* homozygous. (B) Quantitative real-time PCR (qRT-PCR) of *myostatin* mRNA expression normalized to *actin*. Total RNA was isolated from 36 hpf wild-type, *axin1/apc1* and Lef1 morphant embryos. Graphs show that expression of *mstn* is upregulated in Lef1 morphants, corresponding to the *in situ* hybridization with *mstn* probe in bottom panels. Scale bar, 100 µm. (C) Quantitative real-time PCR (qRT-PCR) of *myostatin* mRNA expression normalized to *actin*. Total RNA was isolated from 54 hpf wild-type, *axin1/apc1* and Lef1 morphant embryos. Graphs show that expression of *mstn* is downregulated in *axin1/apc1* embryos and upregulated in Lef1 morphants (D) Western blot on lysates collected from 54 hpf wild-type, *axin1/apc1* and Lef1 morphant embryos. Graph shows upregulation of Mstn in Lef1 morphants. However, there is no significant difference in levels of Mstn protein in *axin1/apc1* embryos versus wildtypes. (E) Misexpression of *mstn* mRNA downregulates the Wnt target gene *lef1* in *axin1/apc1* mutants shown with WISH for *lef1* riboprobe. Scale bar, 100 µm.

As expression profiling in Mstn loss-of-function (LOF) identified modulation of Wnt- pathway components [33], we examined for possible genetic interaction between the two pathways, by using gain and loss of Wnt/β-catenin signaling. To establish a suitable genetic means for analysis of *mstn* mRNA upon loss of Wnt/β-catenin function, we first tested whether morpholino (MO)-mediated knock-down of Lef1 [22], which is upregulated in *axin1/apc1* mutants (Fig. 1B) would rescue their aberrant somitogenesis. Knockdown of Lef1 with 2 ng MO in wild-types resulted in loss of a number of somites (59%, n = 54) at 54 hpf (Fig. 7A), suggesting that Lef1 is required for normal somitogenesis. Notably, in 50% of Lef1-MO-injected *axin1/apc1* mutants, the normal number of somites was restored, establishing a mechanistic link between Lef1 hyperactivity and somite loss (Fig. 7A).

To test whether GOF and LOF of Wnt/β-catenin signaling affected the levels of *mstn* RNA, we employed quantitative real-time PCR to quantify expression of *mstn* mRNA in *axin1/apc1* mutants versus Lef1-morphants at different developmental times. The data showed that reduced Wnt/β-catenin signaling in Lef1 morphants resulted in upregulation of *mstn* mRNA (Fig. 7B, C), whereas hyperactivity of the pathway in the *axin1/apc1* mutants led to a slight *mstn* downregulation at 36 hpf (Fig. 7B), and a more significant downregulation at 54 hpf (Fig. 7C). We then analyzed the level of Myostatin protein at 54 hpf. Predictably and in agreement with the qPCR data, the level of processed mature monomeric Myostatin protein was significantly induced in Lef1-morphants suggesting that reduced Wnt signaling through Lef1 leads to de-repression of Myostatin. Surprisingly, in *axin1/apc1* embryos, the level of Myostatin protein is similar to that of wild-type embryos, suggesting that subtle increase of *myostatin* mRNA in the Wnt/β-catenin GOF context, does not translate into an increase in the stable protein (Fig. 7D). Why and how this mechanistically takes place need further investigation. As *lef1* was upregulated in the somites of *axin/apc* mutants at 30 hpf, we tested whether misexpression of *mstn* would alter this Wnt/β-catenin downstream target gene. Misexpression of *mstn* downregulated ectopic *lef1* in the mutants as well as slightly downregulating *lef1* expression in wildtype siblings (Fig. 7E) showing genetic interaction between Mstn and Wnt/β-catenin signalling and probably underlying the mechanism of muscle fiber rescue. To corroborate possible interaction between the two pathways we performed *in silico* analysis to identify putative TCF-binding elements (TBE) in *myostatin* promoter, as have been identified in the promoters of Wnt target genes [34]. Indeed, we found 3 putative TBE (NNCAAAG) within a 2.8 kb sequence upstream of the myostatin gene at positions -2790, -2389 and -1578 (data not shown) opening up the possibility of a direct interaction between Wnt and Myostatin.

Together, these data suggest an existence of a genetic interaction between Wnt/β-catenin and Mstn in myofibrillogenesis possibly existing as a negative feedback loop. We propose a model describing regulation of fast muscle fiber growth and maintenance during secondary myofibrillogenesis with respect to Mstn and Wnt signaling (Fig. S4).

## Discussion

Most zebrafish mutants such as *after eight (aei)* and *deadly seven (des)* that harbor mutations in genes of the Delta-Notch pathway [35] display a reduction in somite numbers secondary to patterning. Together with Delta-Notch signaling, Wnt/β-catenin signaling contributes to somite clock establishment, mediating early somite patterning [36], [37]. The unique phenotype of the *axin1/apc1* mutant is characterized by normal somite patterning followed by a gradual loss of approximately 10 most caudal somites. Our data show that this somite loss, resulting from sustained, ectopic and hyperactive Wnt/β-catenin, is secondary to cell fate alterations, ultimately leading to hypertrophic and degenerative fast muscle fibers. The hyperactive Wnt signal is transduced mainly through Lef1 and leads to an imbalance between proliferation and differentiation in the myotome. The late activation of Wnt/β-catenin in wild-type embryos by treatment with LiCl phenocopies the fast muscle fiber hypertrophy and degeneration observed in *axin1/apc1* embryos. This corroborates the notion that the mutant muscle fiber phenotype arises independently and is subsequent to normal somite establishment, providing an opportunity to decouple roles of Wnt/β-catenin in myofibrillogenesis versus somite patterning.

High Wnt/β-catenin activity is required for somite patterning, as well as for proliferation i.e. expansion of the Pax3/7+ pre-myogenic progenitor compartment. Pax3 and Pax7 transcription factors, that mark the pre-myogenic progenitors in the developing dermomyotome [38] and satellite cells in the adult muscle, positively regulate cell proliferation [39], [40]. Wnt/β-catenin has been implicated in induction of Pax3/7+ precursors in cell culture systems [41], [42]. A high Wnt/β-catenin activity is probably also required for proliferation of differentiating myoblasts. Our data suggest that the sustained upregulation of *myoD* and *myogenin* in *axin1/apc1* reflects propensity towards differentiation of hyperproliferating pre-myogenic Pax3/7+ precursors being consistent with the known role of Wnt/β-catenin signaling in myogenic differentiation in several systems [43], [44].

We show that hyperproliferative fast muscle fibers underlie the fast muscle fiber degeneration in the *axin1/apc1* mutants, as partial inhibition of proliferation restored to near normal impaired cell survival and fast muscle fiber hypertrophy. The hypertrophic muscle fibers in *axin1/apc1* embryos indicate that a myotomal cell population(s) hyperproliferates and differentiates, thus resulting in an increase in the mass of the muscle fiber. Simultaneously, conflicting instructions to myoblasts to undergo premature differentiation likely leads to apoptosis. To our knowledge, Wnt/β-catenin has as yet not been implicated in muscle hypertrophy *in vivo*. *Ex vivo* studies of the adult muscle reveal the synergistic effect of insulin and Wnt/β-catenin in causing myotube hypertrophy [45]. In addition, it has been shown that Wnt/β-catenin is upregulated in overload-induced hypertrophy of the adult muscle [46]. As a conserved transcriptional hierarchy is thought to regulate the myogenic differentiation in embryos and adults [47], these reported data may be extrapolated to the developing myotome.

Several *in vitro* and *in vivo* studies showed that Mstn overexpression prevents proliferation and differentiation of muscle precursors by inducing expression of the cell cycle inhibitor p21^CIP/WAF^, while endowing muscle progenitors with competence to respond to signals favoring muscle differentiation [32]. We showed that simultaneous knockdown of Lef1 and Myostatin, resulted in a hypertrophied muscle fiber, similar to knockdown of Myostatin. This suggests that Wnt/β-catenin signaling could lie upstream of the Mstn regulatory pathway, as knockdown of Lef1 is unable to rescue the myofiber phenotype (Fig. 6), while misexpression of *mstn* partially rescues the fast muscle hypertrophy in *axin1/apc1* embryos (Fig. 5A). Thus, Wnt/β-catenin might mediate sustained proliferation of muscle progenitors by repressing *mstn*. However, there is also the possibility that the rescue of myofiber growth is non-specific and Myostatin might work independently of and/or in parallel with Wnt/β-catenin signaling in regulating myoblasts proliferation and differentiation. We favor the possibility of a Wnt/β-catenin-Mstn negative feedback loop, as our experimental evidence points towards a specific interaction, direct or indirect, between Wnt/β-catenin signaling and Myostatin as follows: (1) We observe an upregulation of *mstn* RNA transcripts and protein upon Lef1 knock-down which may reflect release from repression of *mstn* by Wnt/β-catenin (2) The downregulation of *lef1* mRNA expression upon *mstn* misexpression in *axin1/apc1* and wildtype embryos suggests a negative feedback loop between Wnt/β-catenin and *mstn*, likely reflecting the mechanism that underlies phenotype-rescuing capacity of Lef1; (3) The identification of 3 putative TBE within a 2.8 kb region upstream of the Myostatin ATG start site opens up a possibility of a molecular interaction between Mstn and Wnt/β-catenin signaling. However, Wnt/β-catenin could also mediate repression of Mstn indirectly, through induction of its direct target follistatin [48] that is a known negative regulator of Mstn [49]. Whether and how this genetic hierarchy regulating myofibrillogenesis translates into direct molecular interactions is an important avenue for further research.

Unlike mice expressing dominant negative Mstn, which equally affects both fast and slow muscle fibers, the *axin1/apc1* embryo exhibits different phenotypes with both slow and fast muscle fibers. Although hypertrophy and hyperplasia is observed in the slow muscle fibers at 36 hpf, there is a reduction in the total amount of slow muscle myosin RNA. We speculate that the lack of quantitative differences observed in the slow muscle fibers could be due to the fact that slow muscle fibers only make up a small portion of the myotome. Therefore, a small increase of slow muscle myosin is not quantifiable by qPCR. On the contrary, the fast muscle fibers exhibit muscle fiber degeneration and disorganization at 36 hpf, and at 54 hpf, they appear hypertrophic (Fig. 2A). This is confirmed by a significant increase of the total amount of fast muscle myosin at 54 hpf (Fig. 2B). The fast muscle hypertrophy is likely to reflect a compensatory response to decreased muscle stability. Significantly, it is only the fast muscle fibers that degenerate in response to hyperactive Wnt/β-catenin signaling even prior to overt hypertrophy. In agreement to the upregulation of fast muscle myosin in *axin1/apc1* embryos, it has recently been shown in cattle that knock-out mutations in myostatin result in preferential downregulation of fast 2× myosin heavy chain [50]. Consistently, *mstn*, which we showed is affected by Wnt/β-catenin pathway is predominantly found in fast twitch muscle [51]. This study opens up a prospect to unravel the poorly understood difference in regulation of maintenance and growth of secondary slow versus fast muscle fibers.

Although our work showed that Mstn negatively regulates Wnt/β-catenin, it is very likely that there is an involvement of other signals that mediate timely and dosage-regulated restriction of the Wnt/β-catenin pathway, thereby safeguarding myofibrillogenesis and regulated muscle growth. The pathogenetic mechanism of the muscle hypertrophy in muscle degenerative diseases is still unclear. Our data, implicating a possible role of Wnt/β-catenin signaling in interaction with Mstn and p21^CIP/WAF^, which have been shown to be important in muscle diseases, might pave a way to approaching muscle diseases from a novel angle.

## Materials and Methods

### Zebrafish embryos

Zebrafish embryos were raised and staged as previously described[52]. *apc^CA50a/CA50a^* is a lethal recessive zygotic mutation identified in a three generation forward mutagenesis screen [25] according to standard mutagenesis protocol. *axin/mbl*(tm13) is a recessive lethal zygotic mutant obtained in the large scale Tubingen screen[24]. *axin1/apc1* compound mutants were generated from crossing *apc^CA50a/CA50a^* with *axin/mbl*(tm13).

### Fish/embryo genotyping

To verify phenotype/genotype correspondence, nested PCR was performed to amplify the template. First amplification was done using outer primer pair apc1forward(1)-apc1reverse(4) for identification of *apc1* mutants, and outer primer pair axin1forwards(1)-axin1reverse(4) for identification of *axin1* mutants. Second amplification for *apc1* and *axin1* was done using primer pair apc1forward(2)-apc1reverse(3) and primer pair axin1forward(2)-axin1reverse(3) respectively. Primer sequences are as follows: apc1forward(1) 5′-GTGCCTTAGAGGTGCAGAAG-3′, apc1forward(2) 5′-GCAGTGTCCTTGTGGTTATG-3′, apc1reverse(3) 5′-TGCCTTTACACATTGGTGAG-3′, apc1reverse(4) 5′-CACAATCCTAACAAGCCATTC-3′, axin1forward(1) 5′-ATGTGTCCTCCATTTGTCTG-3′, axin1forward(2) 5′-TTTGTCTGTCCACATACCTG-3′, axin1reverse(3) 5′-ACACCAGGAAATTCATCCAG-3′, axin1reverse(4) 5′-GATGCTCCTTCATTCCAAAC-3′. DNA sequencing was performed using apc1forward(2) and axin1forward(2) to identify the specific genetic mutations as described previously [24], [25].

### In situ hybridization and immunohistochemistry

Whole-mount in situ mRNA hybridization (WISH) was carried out as previously described[53]. Embryos were fixed in 4% paraformaldehyde (PFA) overnight at 4°C and digoxigenin-tagged probes were made with Roche labeling mix to TOPdGFP, *myoD, myogenin, lef1*, and *titin*. For *mstn*, exonic fragments were generated with the primers: T3mstn(f) 5′-ATTAACCCTCACTAAAGGGAGAATGAACATGCCACCACAGAA-3′ and T7mstn(r) 5′-TAATACGACTCACTATAGGGAGATAATCCAGTCCCAGCCAAAG-3′, and digoxigenin-tagged probes were made. Embryos were fixed for antibody staining with 4% PFA or Carnoy's and whole-mount immunohistochemistry was performed according to Du *et al.* [54], using primary antibodies A4.1025 (Developmental Studies Hybridoma Bank) 1∶20, Eb165 (Developmental Studies Hybridoma Bank) 1∶250, Pax3/7 1∶20 (gift from Prof. N. Patel), PH 3 (Upstate Biotechnology #06570) 1∶1000, MyoD 1∶250 (Santa Cruz, C-20, sc-302). Appropriate secondary antibodies were used at 1∶200. Immunohistochemistry was analyzed at the level of yolk extension where there is minimal muscle degeneration, unless otherwise stated, as caudal to the yolk extension there is massive apoptosis.

### Phalloidin staining

Phalloidin-TRITC (Sigma) staining (1∶50) was performed at room temperature overnight. Muscle fibers were analyzed at the level of the yolk extension where there is minimal muscle degeneration, as caudal to the yolk extension there is massive apoptosis.

### Microinjection of mRNAs and morpholinos (MO)

Morpholino antisense oligonucleotides were obtained from Gene Tool (Philomath, OR): *zflef1* (ATG) 5′-CTCCTCCACCTGACAACTGCGGCAT-3′ [22] and zMstn (ATG) 5′-TGCATGTTCCAAGGCGTGCTAAAGG-3′. We validated effectiveness of Mstn morpholino by showing its capacity to knock-down the protein (Fig. 7D). Capped synthetic mRNA was prepared from pCS2+ constructs encoding zebrafish *mstn* (gift from L.D. Valle) or human p21^CIP/WAF^ (gift from C.J. Weijer) using the mMessage mMachine kit (Ambion), and injected into one-cell stage embryos using a microinjector (World Precision Instruments). A concentration range of 2.5–100 pg of mRNA was injected into one-cell stage embryos to test for viability and effect, and the concentration which had only a subtle effect on wild-type embryos was selected. For *axin1/apc1* rescue experiments, 5 pg of *mstn* or p21^CIP/WAF^ mRNA was used.

### Cell quantification and imaging

Fluorescent labelings were imaged using a Leica TCS SPE confocal microscope. For each set of experiments, all laser and software settings were standardized. Images from each embryo were cropped in Volocity (Improvision) to exclude the neural tube. Cell counts in the somites were done manually from a z-stack of the whole somite. For each set of experiments, cells were counted and imaged at the first four somites of the yolk extension, unless stated otherwise. For quantification of Pax3/7+ pre-myogenic progenitor cells, only weakly labeled Pax3/7+ nuclei were counted as previously described [38]. Digital pictures of WISH embryos were obtained using the Zeiss Axioplan Stereomicroscope (comparable available microscope is Zeiss Axio Imager) equipped with a Leica digital camera and were adjusted for brightness and contrast using Adobe Photoshop 7.0.

### Western blot and quantification

Embryos (54 hpf) were dechorionated, deyolked in deyolking buffer (5 mM KCl, 10 mM D-glucose in PBS), and lysed by sonification for 15 seconds in 50 mM Tris pH 7.5, 150 mM NaCl, 1 mM EDTA, 1% NP-40, 0.1% sodium deoxyocholate and protease inhibitor cocktail (Complete mini, Roche). An equivalent of 12 embryos per lane was fractionated by 17.5% SDS-PAGE gel and blotted semi-dry to PVDF membrane (Millipore). Membranes were stained with Coomassie blue stain to verify loading. Membranes were blocked in blockbuffer (50 mM Tris-HCL, 150 mM NaCl, 0.25% gelatin, 0.5% Triton X-100, pH 7.4) and incubated overnight at 4°C with rabbit anti-Myostatin antibody (AB3239, Millipore, 1∶2500), washed 3×10 min with 100 mM Tris HCl pH 7.5, 0.1% Tween-20 and incubated for 1 h at RT with secondary horseradish peroxidase conjugated anti-rabbit IgG antibody (#554021, BD Transduction Laboratories, 1∶10000), followed by enhanced chemiluminescence (Sigma Aldrich). For actin-loading control, membrane was stripped in 62.5 mM Tris HCl pH 6.8, 2% SDS, 0,14% b-mercaptoethanol, blocked in TBS-0.05% Tween +5% milk and incubated with rabbit anti-actin antibody (A5060, Sigma Aldrich, 1/5000) in TBS-0.05% Tween +2% milk overnight at 4°C, followed by HRP-conjugated anti-rabbit IgG antibody in TBS-0.05% Tween for 1 h at RT, and developed by enhanced chemiluminescence. The film was scanned with GS-800 Calibrated Densitometer (BioRad) and quantitated with Quantity One 4.6.7 program.

### Lithium chloride treatment

LiCl treatment (0.3 M) was repeated twice on the same clutch of embryos for each of the 3 developmental intervals: (1) Early: LiCl treatment pulse for 40 minutes at tailbud and again at 16 hpf, (2) Mid: LiCl treatment pulse for 40 minutes at 16 hpf and again at 24 hpf, and (3) Late: LiCl treatment pulse at 24 hpf and again at 30 hpf. Embryos were washed 3 times in between treatments. Upon treatments, embryos were fixed at 36 hpf, and stained with Phalloidin to visualize all muscle fibers.

### HUA treatment

Embryos were cultured in both 75 µM aphidicolin with 0.25% DMSO (Sigma-Aldrich) and 20 mM hydroxyurea (Sigma-Aldrich) from 24 hpf to 54 hpf. Embryos were then fixed for further experiments.

### BrdU labeling

For BrdU labeling experiments, embryos (16 hpf, 28 hpf, 36 hpf) were dechorionated and placed in 10 mM BrdU with 15% DMSO on ice for 1 hour. After pulsing, embryos were washed in embryo medium several times and incubated at 28°C for 12 hours. Embryos were then fixed with 4% PFA and immunohistochemistry was performed as above, with incubation in 2 N HCl for 1 hour prior to blocking.

### RNA isolation and qRT-PCR

For experiments in Figure 2B, total RNA was isolated from 36 hpf (n = 40) or 54 hpf (n = 40) wild-type and 36 hpf *axin1/apc1* (n = 40) or 54 hpf (n = 40) *axin1/apc1* embryos. ΔΔCT of wild-type was set as 1 for both 36 hpf and 54 hpf, and corresponding values for *axin1/apc1* 36 hpf and *axin1/apc1* 54 hpf were normalized to this wild-type. For experiments in Figure 7B and C, embryos were injected with 2 ng Lef1-MO. At 36 hpf, 40 of each wild-type, *axin1/apc1* homozygous and lef1-MO injected embryos were collected. Total RNA extraction and purification was performed using standard Trizol and isopropanol precipitation. cDNA synthesis was performed using hexamers and M-MLV Reverse Transcriptase. Concentration of purified cDNA was measured with Nanodrop. 50 ng cDNA was used for each set of primers. Transcript levels of *myhz2, myhz5*, *actin* and *mstn* were quantified by real-time PCR using iQ™ SYBR® Green Supermix (Bio-Rad) on an iCycler iQ Real-Time PCR Detection System (Bio-Rad). Results were expressed as a relative ratio to the housekeeping gene actin according to a mathematical method as described [55]. Primer sequences are as follows: mstn(F) 5′-GATTAACGCATATGACGCGAAG-3′, mstn(R) 5′-ACAGTGAGAGGGTACCTGCAG-3′, myhz2(F) 5′-ACAGTTTTTCAACCACCACATGTT-3′, myhz2(R) 5′- AATGCAAGCGGCCAAGTC-3′, myhz5(F) 5′- GCTGGAGAATGAGGTGGAGTTG-3′, myhz5(R) 5′- AGTCTGGTAGGTGAGCTCCTTGA-3′, ActinControl(F) 5′-CAACAGGGAAAAGATGACACAGAT-3′, ActinControl(R) 5′-CAGCCTGGATGGCAACGT-3′. Accession numbers for *mstn* is NM_131019, *myhz2* is NM_152982, *myhz5* is AY333451 and *actin* is AF025305. Triplicates were carried out for each amplification.

### Statistical analysis

Shapiro-Wilk normality test was performed with SPSS 16.0. All data followed normal distribution, with the exception of wild-type DMSO controls in Fig. 3d. Unpaired two-tailed student's t-test was performed using SPSS 16.0. For wild-type DMSO controls in Fig. 3d, where no normal distribution was observed, non-parametric Mann-Whitney test was used. All significant differences (p<0.05) are marked with an asterisk (*) and highly significant differences (p<0.005) are marked with two asterisks (**). All bars in graphs depict mean values with error bars depicting standard deviations.

## Supporting Information

Figure S1 (7.14 MB TIF)

Figure S2 (1.21 MB TIF)

Figure S3 (4.29 MB TIF)

Figure S4 (1.92 MB TIF)
