# Supplementary figures and images for: Regulation of Slow and Fast Muscle Myofibrillogenesis by Wnt/β-Catenin and Myostatin Signaling
Source: PLoS One. 2009 Jun 11;4(6):e5880. doi: 10.1371/journal.pone.0005880 (PMC2690692; doi:10.1371/journal.pone.0005880)

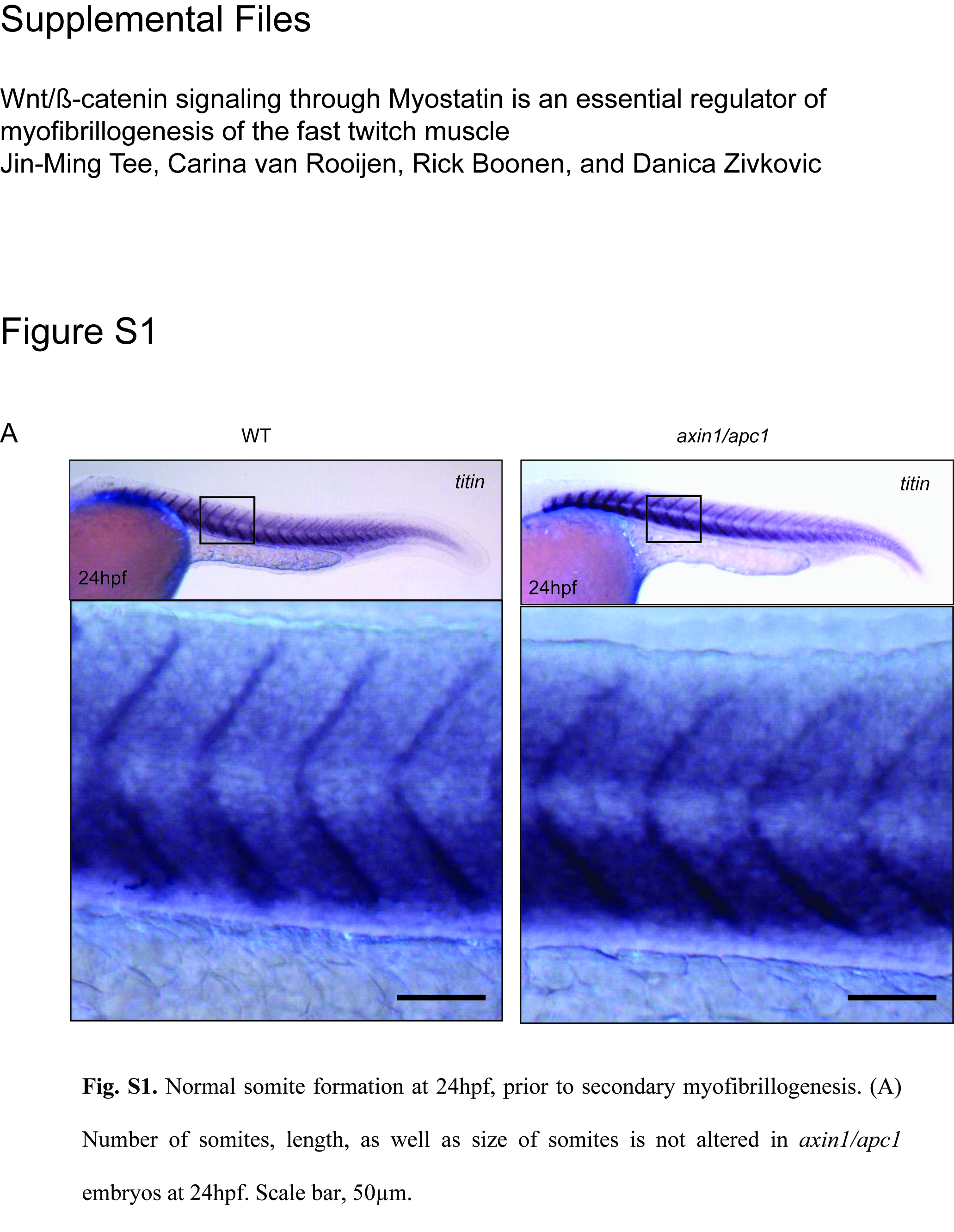

Supplement: Figure S1 — (7.14 MB TIF) [file pone.0005880.s001.tif]

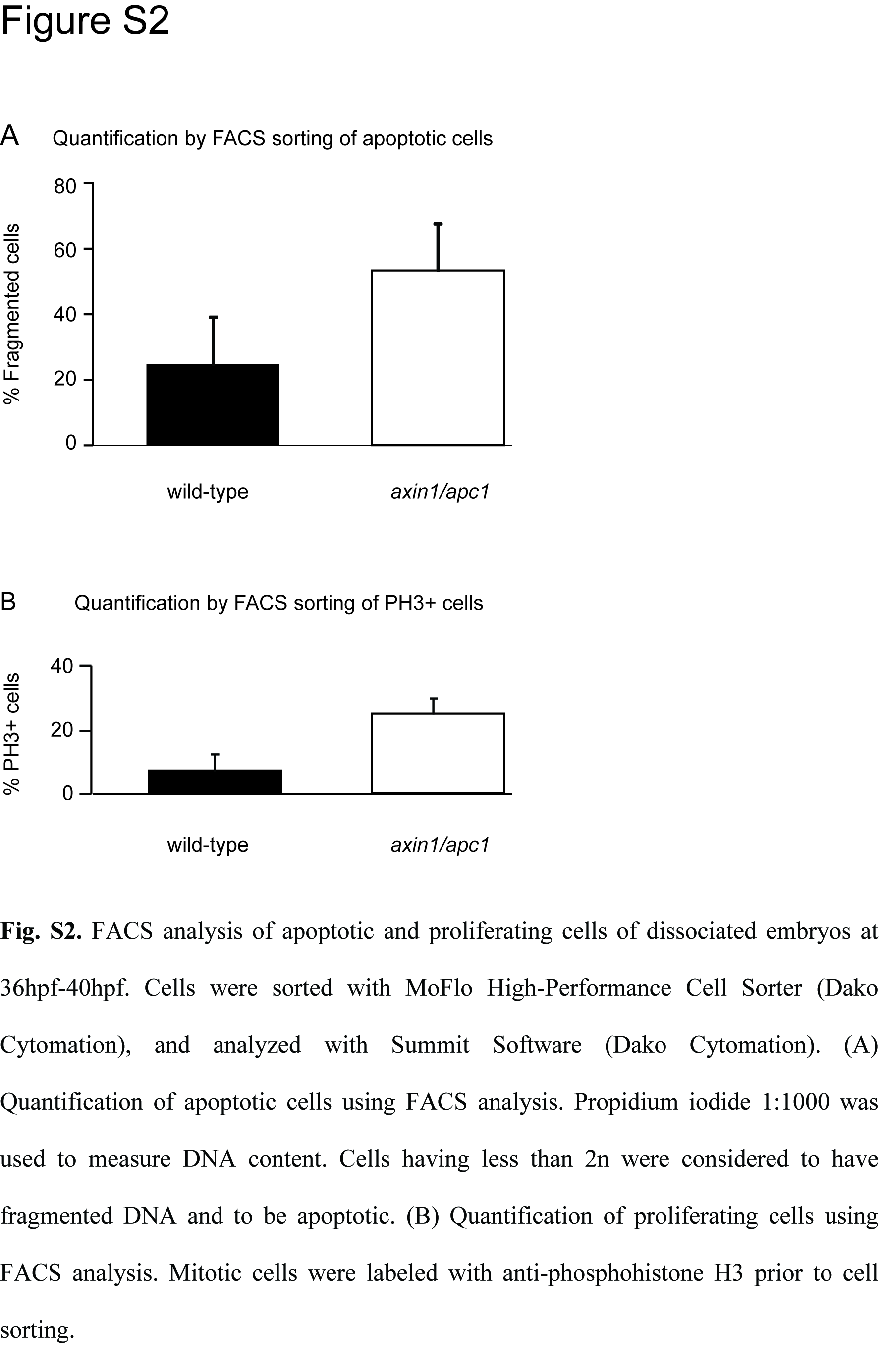

Supplement: Figure S2 — (1.21 MB TIF) [file pone.0005880.s002.tif]

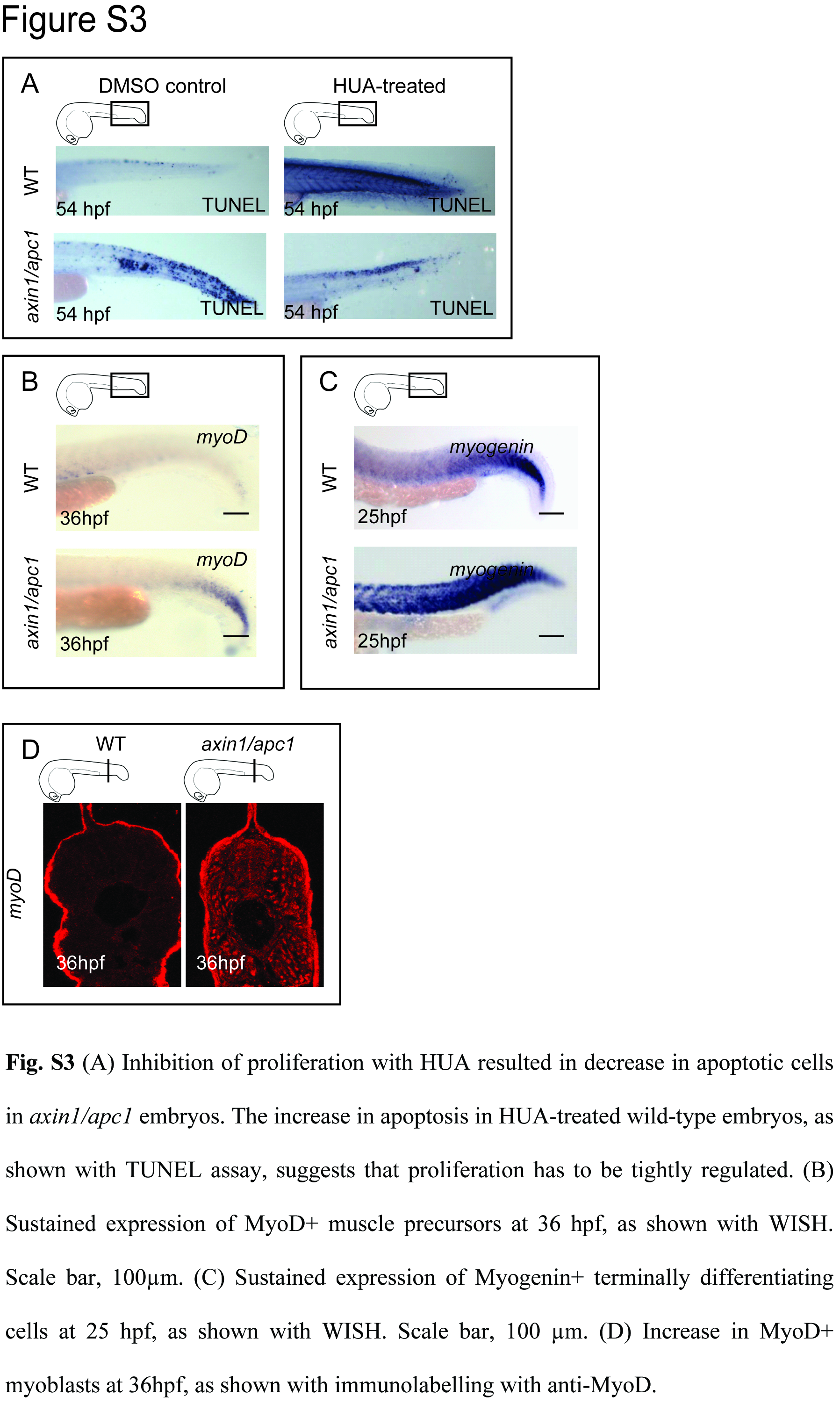

Supplement: Figure S3 — (4.29 MB TIF) [file pone.0005880.s003.tif]

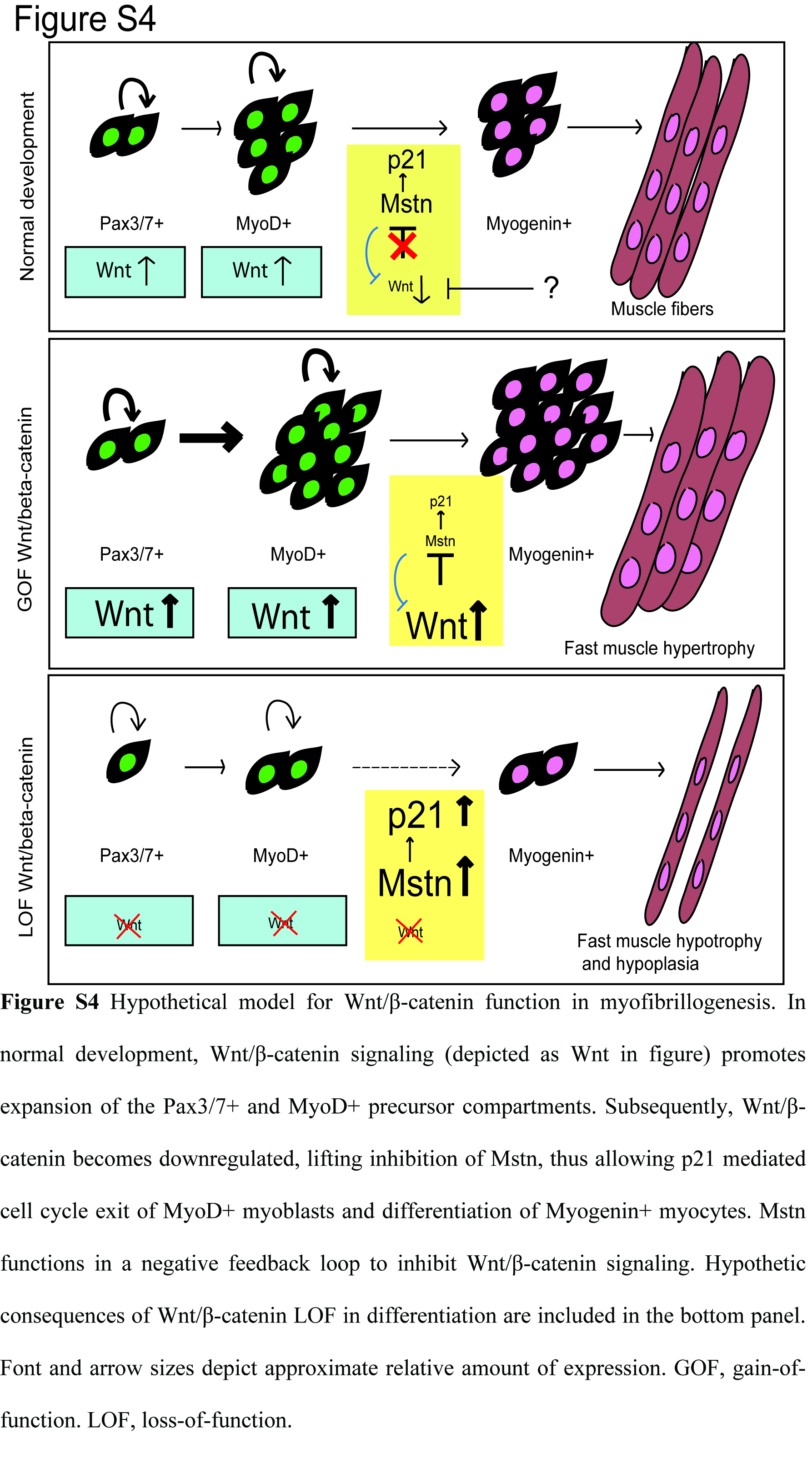

Supplement: Figure S4 — (1.92 MB TIF) [file pone.0005880.s004.tif]
